# Applying the Mini-Open Anterolateral Lumbar Interbody Fusion with Self-Anchored Stand-Alone Polyetheretherketone Cage in Lumbar Revision Surgery

**DOI:** 10.1155/2016/1758352

**Published:** 2016-11-03

**Authors:** Lei Kuang, Yuqiao Chen, Lei Li, Guohua Lü, Bing Wang

**Affiliations:** Department of Spinal Surgery, The Second Xiangya Hospital of Central South University, Changsha, Hunan, China

## Abstract

The author retrospectively studied twenty-two patients who underwent revision lumbar surgeries using ALLIF with a self-anchored stand-alone polyetheretherketone (PEEK) cage. The operation time, blood loss, and perioperative complications were evaluated. Oswestry disability index (ODI) scores and visual analog scale (VAS) scores of leg and back pain were analyzed preoperatively and at each time point of postoperative follow-up. Radiological evaluation including fusion, disc height, foraminal height, and subsidence was assessed. The results showed that the ALLIF with a self-anchored stand-alone PEEK cage is safe and effective in revision lumbar surgery with minor surgical trauma, low access-related complication rates, and satisfactory clinical and radiological results.

## 1. Introduction

Posterior approaches, such as posterior lumbar interbody fusion (PLIF) and transforaminal lumbar interbody fusion (TLIF), are commonly used in revision lumbar surgery partially because of their advantage of directly removing problematic implants and fractured screws and rods [1, 2]. Solid lumbar fusion requires internal fixation to help achieve immobilization. However, these approaches also increase the risk of damaging the posterior muscular elements, leading to long-term back pain [1]. In addition, extensive adjacent-level facet joint violations have been reported with posterior revision surgery, which theoretically leads to instability of the upper adjacent level and may accelerate adjacent segment degeneration (ASD) [3, 4]. Significantly higher incidental durotomy rates have been found in posterior revision surgery than in primary surgery due to scar tissue adhesion [5]. Anterior lumbar interbody fusion (ALIF) is an alternative approach when dealing with ASD, recurrent disc herniation, cage migration, and pseudarthrosis. It provides direct access to the vertebral column and allows more extensive decompression of the disc space and better end plate preparation for arthrodesis, while simultaneously restoring disc height and correct lumbar kyphosis [6]. Moreover, ALIF avoids posterior muscle trauma, adjacent-level facet joint violation, and acceleration of ASD [6, 7]. Nevertheless, access-related complications have been documented, such as urethral injury, bowel perforation, incisional hernia, neurological injury, ileus, and retrograde ejaculation in men, with vascular injury being the most disastrous [2, 7–11]. The transpsoas exposure of extreme lateral interbody fusion (XLIF) reduces manipulation of the aorta and vena cava; hence, the incidence of vascular injury is lower [12–15]. However, this approach is associated with access-related thigh symptoms, such as numbness, pain, and weakness, resulting from injury of the lumbar plexus or motor nerves, especially when the L4/5 level is involved [16].

Minimally invasive lumbar surgery techniques were first described by Mayer in 1997, which were advocated as an alternative to anterior or posterior approaches for lumbar fusion with less surgical trauma and quicker recovery [17]. This approach used a psoas-preserving access to the lumbar spine via the anterior oblique retroperitoneal approach, but with less invasion of the psoas muscle and lumbar plexus than XLIF. To distinguish this new technique from other minimally invasive ALIF, Silvestre et al. renamed it the oblique lumbar interbody fusion (OLIF) [13]. However, the L5/S1 level can only be achieved through transperitoneal approaches, which provides only indirect decompression. It is still not possible to treat conditions such as recurrent lumbar disc herniation without subsequent posterior surgery, which inevitably increases the surgical trauma.

The recently developed mini-open OLIF allows psoas-preserving access to the lumbar spine via the anterior oblique retroperitoneal approach with less invasion of the psoas muscle and a reduced incidence of lumbar plexus and motor nerve injury [18]. However, this approach allows only a limited operative field, and direct decompression is hard to achieve. Although it has been reported that spinal stenosis could be resolved successfully by indirect decompression, posterior fixation cannot be avoided [18]. In this study, for the first time, an ALLIF using a self-anchored stand-alone polyetheretherketone (PEEK) cage was used to increase the visual field and to facilitate direct decompression. The safety and efficacy of this procedure were also evaluated to investigate whether it could serve as a new alternative to anterior revision surgery after posterior lumbar surgery.

## 2. Materials and Methods

### 2.1. Study Population

Between April 2012 and April 2014, a total of 22 patients who underwent the ALLIF revision surgery and met the following criteria were recruited: (1) initial posterior surgery for lumbar degenerative disc disease or lumbar spondylolisthesis, (2) age between 18 and 65 years, (3) patients with back and/or leg pain after initial surgery who were unresponsive to appropriate conservative treatment, (4) having conditions such as recurrent disc herniation, pseudarthrosis, adjacent segment degeneration, or cage migration confirmed by computed tomography (CT) or magnetic resonance imaging (MRI), and (5) having 24 months or more of follow-up data. Patients with the following criteria were excluded: (1) previous abdominal or anterior lumbar surgery history, (2) posterior scarred adhesion compressing the nerve structure confirmed by medical history or physical or radiological examination, (3) abdominal aortic aneurysm or severe peripheral vascular disease, (4) obesity with BMI ≧ 28 kg·m^2^, and (5) severe osteoporosis. The characteristics of these included patients were listed in Table 1. The mean follow-up time was 24.6 ± 6.7 months. All procedures were performed by the same surgeon (Lü), who has rich experience with anterior lumbar surgery and laparoscopic lumbar surgery for lumbar degenerative disease, deformity, tumor, and infection.

All procedures performed in studies involving human participants were in accordance with the ethical standards of the institutional and/or national research committee and with the 1964 Helsinki Declaration and its later amendments or comparable ethical standards. Informed consent was obtained from all individual participants included in this study.

### 2.2. Surgical Procedure

**Figure 1:**
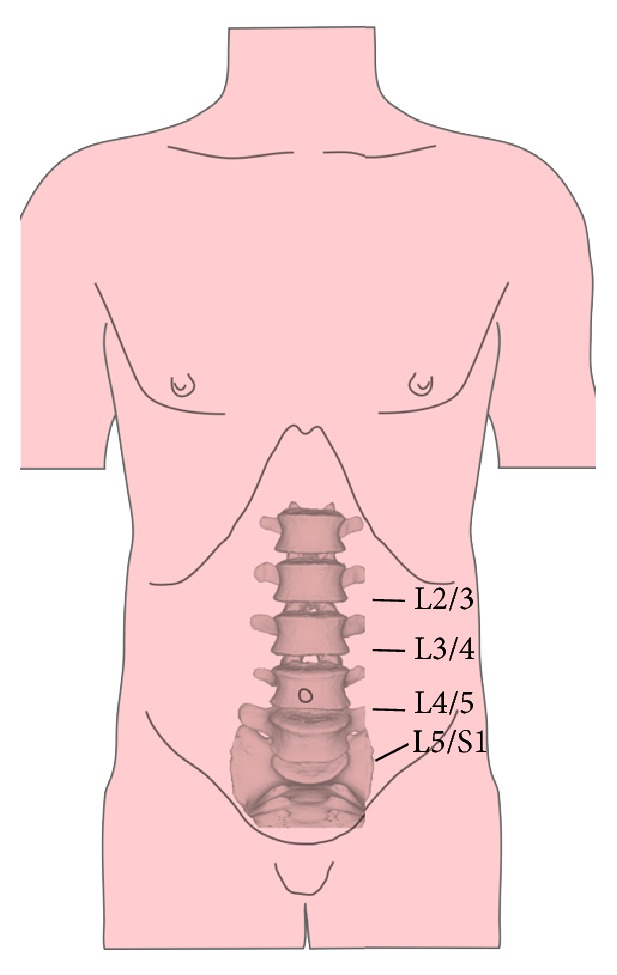
A transverse or oblique 4 to 6 cm skin incision was made on the lateral wall of the abdomen, parallel to the projection of the affected disc level.

**Figure 2:**
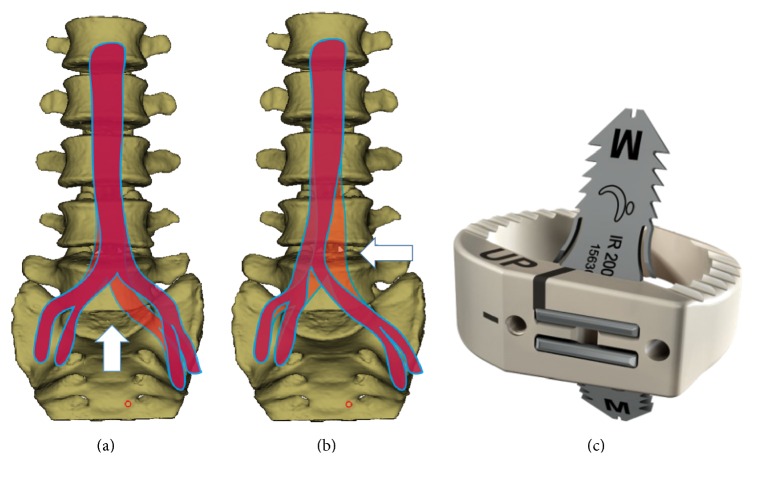
(a) The access to L5/S1 when the aortic bifurcation is high. (b) The access to L5/S1 when the aortic bifurcation is low. (c) The self-anchored PEEK cage we used in the study (ROI-A Oblique, LDR Médical, Troyes, France).

Patients were placed in the supine position. A transverse skin incision of 4 to 6 cm was made on the lateral wall of abdomen, parallel to the projection of the affected disc level (Figure 1). The external oblique, internal oblique, and transverse abdominal muscles were then bluntly dissected. The peritoneal content was mobilized inwardly. Headlights were used to illuminate the operation field. The lateral edges of the iliac artery and the iliac vein were bluntly separated from the spine using gentle, peanut sponge, and fingertip dissection. A hand-held abdominal retractor was placed on the anterolateral part of the spine with vessels and the peritoneal contents retracted medially. For operations at and above L4/L5, the psoas muscle and lumbar plexus were identified and mobilized. Another hand-held abdominal retractor was placed on the lateral side of the spine gently retracting the psoas muscle and sympathetic nerves posteriorly. The intervertebral disc was exposed between the psoas muscle and aorta. For operations at L5/S1, exposure was carried out below the aortic bifurcation or over the shoulder of the aortic bifurcation (between the psoas muscle and left iliac artery) according to the relationship of aorta and the L5/S1 disc, assessed by CTA or MR preoperatively (Figure 2). The operation levels were identified fluoroscopically. After discectomy, a nerve hook was used to explore the lateral recess and posterior edge of the vertebra to confirm complete decompression. Endplate preparation was performed using curettes. The disc space was distracted using a parallel distractor. A proper-sized self-anchored PEEK cage (ROI-A® Oblique, LDR Médical, Troyes, France) (Figure 2(c)) was determined by trials under fluoroscopy. Cages were inserted obliquely into intervertebral space using fluoroscopy after filling with porous bioceramic artificial bone (Dragonbio®, Hubei, China). Once the position of the cage was optimal, two self-guided anchoring plates were inserted into the adjacent vertebrae under fluoroscopy.

### 2.3. Clinical Outcome Measurements

Operative time, blood loss, and intra- and postoperative complications were noted. Clinical outcomes including the Oswestry low back pain disability index (ODI) and visual analog scale (VAS) for back pain and leg pain were measured preoperatively and postoperatively at 3, 6, 12, and 24 months.

### 2.4. Radiological Outcome Measurements

Fusion was identified by the presence of continuous bridging trabeculae at the graft and end plate junction on radiographs or CT scans [19]. Pseudarthrosis was defined when assessment failed to meet the fusion criteria at the last follow-up. Other radiological outcomes (foraminal height, disc height, and subsidence) were measured preoperatively and at 2 days and 3, 6, 12, and 24 months postoperatively. Disc height was defined as the mean value of the anterior disc height and posterior disc height. The foraminal height was determined as the longest distances between the craniocaudal dimensions of the foramen [20]. Subsidence was defined as any compromise of either vertebral endplate visible on CT scan or X-ray [21].

### 2.5. Statistical Analysis

All statistical analyses were conducted using SPSS version 19.0 software (SPSS Inc., Chicago, IL, USA). Comparisons between the preoperative and postoperative parameters within the groups were performed using a paired* t*-test. A *p* value < 0.05 was considered statistically significant.

## 3. Results

**Figure 3:**
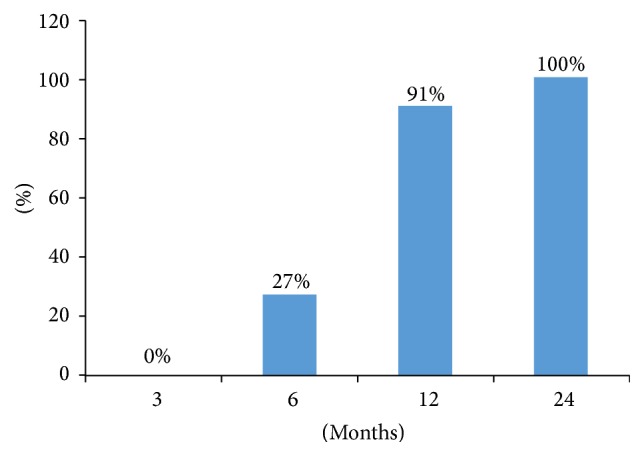
A column diagram demonstrating the fusion rate of the patients at each time point.

Patient characteristics including age, gender, primary surgery, primary operation levels, reasons for revision surgery, and revision levels are summarized in Table 1. There were 13 females and 9 males aged between 48 and 63 years with a total of 27 segments enrolled in this study. There were 7 patients excluded for meeting the exclusion criteria. The average age was 55.4 ± 5.5 years. Of all these 22 patients, 19 had posterior instrumentation in their previous surgery. And 7 of them experienced the failure of the posterior instrumentation before the revision surgery. The single-level cases included 9 cases at L4/5, 2 cases at L3/4, and 6 cases at L5/S1; 5 cases were with two levels. Only one patient suffered from peritoneal rupture during the exposure. No other perioperative complications were found. Four patients with 4 operated levels suffered cage subsidence without clinical symptoms (Table 1). Fusion was achieved in all patients (Figure 3).

The average operating time was 68.6 ± 22.9 minutes, and the average estimated blood loss was 85.4 ± 34.7 mL. As shown in Table 2, the VAS back pain score decreased from 5.8 ± 1.5 preoperatively to 2.2 ± 0.9, 2.4 ± 1.0, 2.4 ± 0.8, and 2.3 ± 0.9 postoperatively at 2 weeks and 3, 6, 12, and 24 months, respectively (*p* < 0.05). The average VAS leg pain score also decreased from 5.3 ± 1.6 preoperatively to 2.0 ± 1.3, 2.2 ± 1.3, 2.3 ± 1.0, and 2.1 ± 1.1 at 3, 6, 12, and 24 months, respectively (*p* < 0.05). The average preoperative ODI score was 42.7 ± 12.6%. Similarly, at 3, 6, 12, and 24 months after surgery, the postoperative ODI scores were significantly decreased to 27.5 ± 8.2%, 25.5 ± 8.5%, 23.8 ± 6.8%, and 23.4 ± 6.1%, respectively (*p* < 0.05).

The average foraminal height was 15.8 ± 3.4 mm before surgery and increased postoperatively to 19.4 ± 2.8 mm at 2 days, 19.0 ± 3.1 mm at 3 months, 18.7 ± 2.7 mm at 6 months, 18.5 ± 2.5 at 12 months, and 18.2 ± 2.7 mm at 24 months (*p* < 0.05). The average disc height also increased from 8.6 ± 2.5 mm preoperatively to 12.3 ± 1.5 mm, 11.8 ± 2.2 mm, 11.6 ± 2.3 mm, 11.3 ± 2.3 mm, and 11.0 ± 2.0 at 2 days and 3, 6, 12, and 24 months after surgery, respectively (*p* < 0.05). The results are summarized in Table 3.

## 4. Discussion

Anterior lumbar spinal surgery has been commonly used in conditions that include disc degeneration, trauma, infection, deformity, and tumor with approaches such as ALIF, XLIF, and OLIF [7]. Recently, these anterior approaches were adopted in lumbar revision surgery [5, 22]. Mamuti et al. retrospectively reviewed 35 patients who underwent mini-open retroperitoneal anterior lumbar interbody fusion using self-anchored cage device for the treatment of recurrent lumbar disc herniation following primary posterior instrumentation [23]. Their result showed good clinical and radiological outcomes without complications related to surgical technique and cage device. Furthermore, Mobbs et al. recommended that anterior lumbar interbody fusion could be a salvage technique for pseudarthrosis following posterior lumbar fusion surgery when the chronic low back pain raised by pseudarthrosis was nonresponsive to conservative management [24]. Anterior lumbar interbody fusion could provide a wider implant bed and more meticulous preparations of endplates for arthrodesis, which lead to the high fusion rate theoretically.

The approach-related complications concern most researchers. Bateman et al. performed a systematic review to identify the types and incidence rates of complications associated with various approaches to anterior lumbar spine surgery. The results showed that the overall complication rate was 14.1% with intraoperative and postoperative complication rates of 9.1% and 5.2%, respectively. The most common complications reported were venous injury (3.2%), retrograde ejaculation (2.7%), neurologic injury (2%), prosthesis-related (2%), postoperative ileus (1.4%), superficial infection (1%), and complications classified as “others” (1.3%). Laparoscopic and transperitoneal procedures were associated with higher complication rates, whereas lower complication rates were observed in patients receiving mini-open techniques. A study by Fujibayashi et al. evaluated twenty-eight patients who underwent OLIF for lumbar degeneration disease [18]. Two cases of hip flexor weakness and 6 cases of thigh pain/numbness that resolved spontaneously within 3 months after operation were observed. In our study, no major approach-related complications, such as vascular injuries, ureteral injuries, visceral complication (bowel perforation), ileus, incisional hernia, or retrograde ejaculation, were observed. This suggested that the ALLIF technique is a relatively safe procedure. Other factors attributing to a low complication rate should also be considered. All procedures were performed by skilled surgeons with extensive anterior spinal surgery experience. Preoperative CT angiography was taken to evaluate difficulties in the exposure because vascular injuries were prone to occur in presence of anatomical variation, or with surrounding scar tissue [25]. Because micromotion in the bone-graft interface is believed to be one of the main reasons for pseudarthrosis and cage subsidence [1, 26], additional pedicle screws have been used to provide sufficient primary stability after mini-open OLIF [18]. However, the self-anchored PEEK cage we used (ROI-A Oblique, LDR Médical, Troyes, France) has two integrated self-locking clips bridging the index levels which was designed to provide stronger lumbar stability, avoid the motions between the adjacent vertebral bodies, and promote solid fusion. A biomechanical test revealed that the self-locking stand-alone cage could provide immediate stability that was equivalent to that with anterior plate or posterior pedicle screw fixation [27]. Clinical studies have also demonstrated that a high fusion rate (90.6% to 97.3%) with good clinical results could be achieved using these self-anchored designed stand-alone cages [3, 25, 28]. In our study all patients achieved solid fusion at the last follow-up which supported the hypothesis that these self-anchored stand-alone cages could provide immediate stability after surgery and reach high fusion rate.

**Figure 4:**
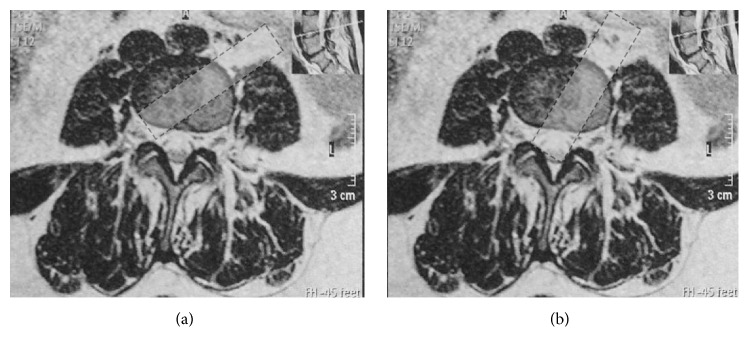
(a) Only indirect decompression can be achieved in OLIF because of the limited operation angle and field. (b) A wider operation angle and space can be provided for direct decompression in ALLIF with the skin incision placed closer to the middle line of the abdomen.

**Figure 5:**
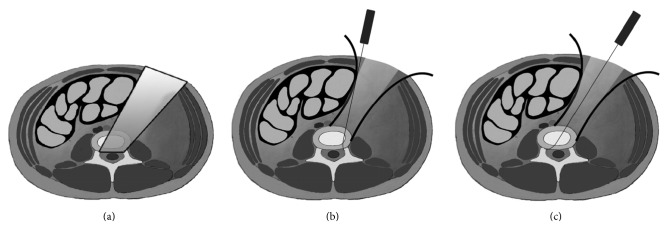
(a) The ideal operation field for direct decompression. ((b) and (c)) The operation field of the ALLIF.

In our ALLIF, a transverse skin incision placed closer to the middle line of the abdomen was made on the lateral wall at the outer rim of abdominal rectus muscle, compared with the typical incision for OLIF. This slight adjustment provides a wider visual and operative field (Figure 4). All discectomy procedures could be performed under direct visualization, which made it possible to decompress the neurological structure bilaterally without damaging the nerve element or dural sac, thus avoiding posterior decompression surgery. A nerve hook could be used to explore the lateral recess and posterior edge of the vertebra to confirm complete and thorough decompression, which could not be achieved in the OLIF because of the operation angle (Figure 5). The indirect decompression in OLIF is achieved by disc distraction and not by the removal of the compressing element. The better operation angle in ALLIF also makes it possible to access every L5/S1 level, even in patients with a high-riding pelvis, which may not be possible in OLIF. Moreover, the cage was easily inserted obliquely along this access angle, which largely reduced the manipulation of the aorta and vena cava, decreasing the risk of vascular injury compared with ALIF. Retraction of vascular structures throughout an entire procedure was blamed for the increase in vessel injuries and thrombotic events in OLIF [7, 29]. Therefore, we used hand-held abdominal retractors instead of self-retaining retractors to expose the discs, for they could be released intermittently to minimize the risk of vascular thrombosis. Besides, in the traditional OLIF, the access to disc of L5/S1 was below the aortic bifurcation. In our ALLIF, the access to L5/S1 disc could be below the aortic bifurcation or over the shoulder of the aortic bifurcation (between the psoas muscle and left iliac artery) according to the vascular windows at L5/S1 disc assessed by CTA or MR preoperatively. In an anatomy study by Molinares et al., 31% of MR images of patients (31/100) showed no anterior access to L5/S1 disc. However, in 4 (12.9%) of these 31 MR images, an oblique access to L5/S1 disc was found between the psoas muscle and iliac artery. We also adopted the muscle-splitting approach in our study as the so-called “sliding window” technique described by Mayer [17]. Thus, we could easily expose two discs with a slight increase in skin incision length.

**Figure 6:**
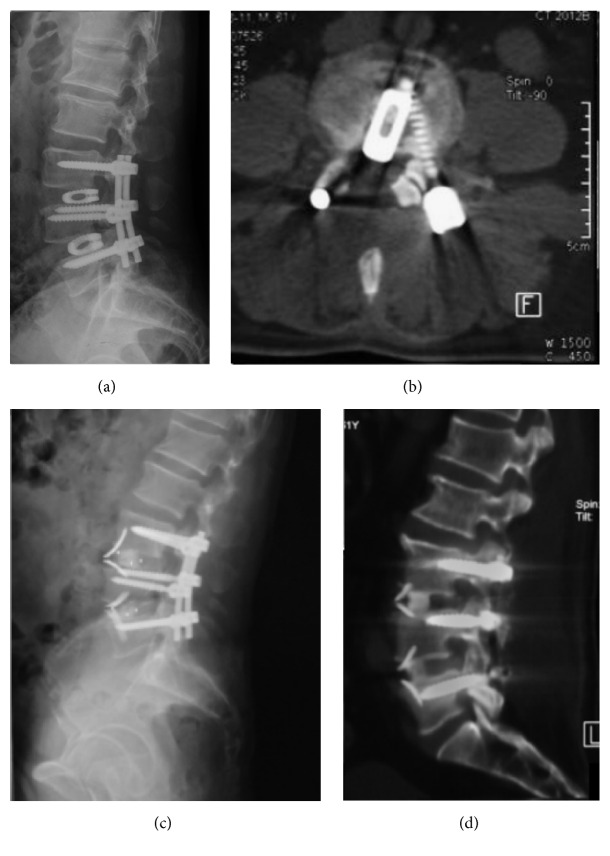
Case presentation. A 61-year-old male patient with previous PLIF surgery at L3-L5 10 months ago was admitted because of the recurrence of back and leg pain. Cage migration was confirmed by both radiograph and CT scan ((a) and (b)). ALLIF revision surgery using a self-anchored cage was performed. Good position of cage levels and satisfactory alignment of the lumbar spine were achieved (c). Fusion was achieved at 12-month follow-up (d).

The patients in our study showed significant improvement in disc height and foraminal height compared with the preoperation status at each time point (Figure 6). In addition, the VAS and ODI scores decreased significantly after surgery compared to baseline. Studies by Siepe et al. [3] and Allain et al. [28] have shown similar results with significant improvement in disc height and foraminal height and decrease in VAS and ODI scores at each time point of follow-up after surgery. Subsidence of the implant into the vertebral endplate may lead to progressive lumbar deformity and recurrence of foraminal stenosis and neurological symptoms, which have been of concern to researchers. The subsidence rate has varied in different studies using a self-anchor stand-alone cage without posterior fixation. In the study by Allain et al., 1 out of 51 analyzed cases experienced subsidence at a 12-month follow-up using cages similar to those we used [28]. Behrbalk et al. reported that 16% (5/32) of cases of subsidence were observed with ALIF using another kind of self-anchor stand-alone cage (SynFix-LR) without posterior instrumentation [30, 31]. A decreased bone mineral density, an increased number of fused segments, damage of the endplate, overdistraction of the surgical segment, and use of oversized cages are thought to contribute to subsidence [32–34]. Beutler and Peppelman Jr. found that most of the subsidence cases happen in the first 3 months postoperatively [32]. Besides, they demonstrated that the cage subsidence is usually accompanied by the appearance of the pseudarthrosis. The long-term micromotion at the nonfused segment damaged the endplate and absorbed the cancellous bone underneath. In the present study, 18.2% (4/22) of patients suffered subsidence and all cases of subsidence were observed before the first 6-month follow-up. Nevertheless, all cases of subsidence reached solid fusion at the last follow-up. Study showed that although the subsidence was not uncommon, the rate of symptomatic subsidence is relatively low. In the study of Le et al., radiographical subsidence occurred in 14.3% (20/140) and the symptomatic subsidence was noted only in 2.1% (3/140) of all patients [33]. In our series, all patients with cage subsidence had no clinical symptoms. Researches demonstrated that the caudal endplate is weaker than the cranial one [33, 34]. Thus the caudal endplate is at higher risk of injury with the stronger cranial endplate usually remaining intact. Similarly, in our series, the damage of caudal endplate was found in all cases with only one case of cranial endplate damage.

This study has several potential limitations. It was a noncontrolled study with a relatively small number of patients, and the inclusion criteria were restrictive. Patients with osteoporosis or with risk factors of access-related complication were excluded, which may have led to an underestimation of the rates of nonunion, subsidence, and access-related complications.

## 5. Conclusion

The ALLIF using a self-anchored stand-alone PEEK cage is a relatively new surgical technique for lumbar revision that provides a wide visual field for operations such as direct decompression. This technique is a safe and effective method in revision lumbar surgery with only limited surgical trauma, low access-related complication rates, and satisfactory clinical and radiological results. The decreased incidence in nonunion and cage subsidence observed may be attributed to the delicate design of this self-anchored PEEK cage.

## Figures and Tables

**Table 1:** Characteristics of 22 patients who underwent revision lumbar surgery using ALLIF with self-anchored stand-alone PEEK cage.

Patient number	Gender	Age	Primary surgery	Reasons for revision surgery	Operation levels	Posterior fixation	Cage subsidence
1	Female	56	TLIF for L4/5 LDH	Implant migration	L4/5	Intact	No
2	Male	61	TLIF for L3/L4, L4/5 LDH	Implant migration	L3/4, L4/5	Intact	No
3	Female	61	TLIF for L4/5, L5/S1 LDH	Pseudarthrosis	L4/5, L5/S1	Breakage of the screw	L4/5
4	Female	49	PLIF for L4/5 LDH	ASD	L5/S1	Intact	No
5	Female	57	TLIF for L5/S1 LDH	ASD	L4/5	Intact	No
6	Female	50	PLIF for L5/S1 LDH	ASD	L4/5	Intact	No
7	Male	52	TLIF for L5/S1 LDH	ASD	L4/5	Intact	No
8	Female	63	TLIF for L4/5 LDH	ASD	L5/S1	Intact	No
9	Male	53	TLIF for L3/L4, L4/5 LDH	Pseudarthrosis	L3/4, L4/5	Breakage of the rod	L4/5
10	Male	48	L5/S1 discectomy	Recurrent disc herniation	L5/S1	None	No
11	Female	42	L4/5 discectomy	Recurrent disc herniation	L4/5	None	No
12	Male	62	L4/5 discectomy	Recurrent disc herniation	L4/5	None	
13	Female	55	TLIF for L5 spondylolisthesis	ASD	L4/5	Intact	No
14	Female	58	Decompression and PLF for L5/S1 LDH	Recurrent disc herniation	L5/S1	Intact	No
15	Male	56	TILF for L4/5 LDH	Pseudarthrosis	L4/5	Breakage of the rod	No
16	Male	60	Decompression and PLF for L4/5 LDH	Recurrent disc herniation	L4/5	Intact	No
17	Female	59	TLIF for L5 spondylolisthesis	Pseudarthrosis at L5/S1 and ASD at L4/5	L4/5, L5/S1	Breakage of the rod	L4/5
18	Female	63	TLIF for L4 spondylolisthesis	ASD	L5/S1	Intact	No
19	Male	52	TLIF for L5 spondylolisthesis	Pseudarthrosis	L5/S1	Screw loosening	L5/S1
20	Female	57	PLIF for L3/4, L4/5 LDH	Pseudarthrosis	L3/4	Intact	No
21	Female	50	TLIF for L3/4, L4/5 LDH	Pseudarthrosis at L4/5 and ASD at L5/S1	L4/5, L5/S1	Intact	No
22	Male	54	PLIF for L3/4, L4/5, L5/S1 LDH	Pseudarthrosis at L4/5	L4/5	Breakage of the rod	No

*PLIF*: posterior lumbar interbody fusion, *PLF*: posterolateral lumbar fusion, *TLIF*: transforaminal lumbar interbody fusion, *LDH*: lumbar disc herniation, and *ASD*: adjacent segment degeneration.

**Table 2:** Clinical outcomes measured by VAS and ODI scores.

	Preop	3 months	6 months	12 months	24 months
VAS back pain	5.8 ± 1.5	2.2 ± 0.9^*∗*^	2.4 ± 1.0^*∗*^	2.4 ± 0.8^*∗*^	2.3 ± 0.9^*∗*^
VAS leg pain	5.3 ± 1.6	2.0 ± 1.3^*∗*^	2.2 ± 1.3^*∗*^	2.3 ± 1.0^*∗*^	2.1 ± 1.1^*∗*^
ODI	42.7 ± 12.6%	25.5 ± 8.5%^*∗*^	23.8 ± 6.8^*∗*^	23.4 ± 6.1%^*∗*^	24.0 ± 6.5%

^*∗*^Statistically significant compared with preoperation (*p* < 0.05).

*Preop*: preoperatively, *3 months*: 3 months postoperatively, *6 months*: 6 months postoperatively, and *12 months*: 12 months postoperatively.

**Table 3:** Radiological outcome measured by disc height and foraminal height (mm).

	Preop	Postop	3 months	6 months	12 months	24 months
Disc height	8.6 ± 2.5	12.3 ± 1.5^*∗*^	11.8 ± 2.2^*∗*^	11.6 ± 2.3^*∗*^	11.3 ± 2.3^*∗*^	11.0 ± 2.0^*∗*^
Foraminal height	15.8 ± 3.4	19.4 ± 2.8^*∗*^	19.0 ± 3.1^*∗*^	18.7 ± 2.7^*∗*^	18.5 ± 2.5^*∗*^	18.2 ± 2.7^*∗*^

^*∗*^Statistically significant compared with preoperation (*p* < 0.05).

*Preop*: preoperatively, *postop*: postoperatively, *3 months*: 3 months postoperatively, *6 months*: 6 months postoperatively, and *12 months*: 12 months postoperatively.
